# Using the Office of Management and Budget (OMB) Clearance Process in Program Planning and Evaluation

**Published:** 2005-12-15

**Authors:** Maurice Martin, Darlene Thomas

**Affiliations:** Centers for Disease Control and Prevention (CDC), Division of Diabetes Translation (DDT); CDC, DDT, Atlanta, Ga

## Program Evaluation and Data Collection

Through well-planned health promotion and education programming, federally funded projects are often leaders in the development and implementation of sound public health practices. In public health practice, program evaluations are used to 1) determine whether program objectives related to health status have been achieved, 2) improve program implementation, 3) increase community support, 4) contribute to a scientific base, 5) provide accountability to community members and other stakeholders, and 6) guide program-specific policy decisions (1,2). To monitor program processes and obtain the evidence necessary to demonstrate a program's effectiveness, some form of evaluation involving data collection is usually completed (3). Evaluation is considered a key component of public health programming (2,4).

Evaluation methodologies vary significantly among programs, as do data-collection techniques. In some evaluations, it is appropriate to use previously collected data that were not originally intended for measuring a program's processes and outcomes. For instance, mortality or disease rates from county vital records or reportable disease records of state health departments can be used to guide certain health promotion programming decisions. However, generally, comprehensive program evaluations involve planned process measures to gauge completeness and quality of activities leading to outcomes in addition to less formal process measures that come about as adjustments to health promotion programming. More formal evaluation measures are characterized as systematic; they have long-term outcome indicator measurements that are planned before the program implementation (2-5).

## The Paperwork Reduction Act (PRA)

Evaluations of public health programs are often conducted at taxpayers' expense because federal agencies are accountable for the quantity and quality of the information resulting from these evaluations. The U.S. public deserves this, and the U.S. Congress demands it (6). However, in response to constituent complaints about unnecessary and often redundant data collections, Congress passed the Paperwork Reduction Act (PRA) in 1980 to help balance the public demand for accountability and the resulting paperwork burden on the public (7). The law was created to ". . . ensure that federal agencies do not overburden the public with federally sponsored data collections," with *burden* being defined as ". . . the total time, effort, or financial resources expended by persons to generate, maintain, retain, or disclose or provide information" (6). In 1995, more reductions were proposed, and the PRA was amended. Congress mandated a 25% reduction in burden in the 3 years immediately following the amendment's passage (8,9).

Compliance with the PRA is required whenever a federal agency sponsors a data collection by using identical questions, using identical reporting or record-keeping requirements, or asking respondents to provide the same level of information on the same subject involving 10 or more respondents in a 12-month period (7,10). The law applies to all federal employees, contractors, people in cooperative agreements, and anyone else who asks the public for information for the purpose of research, public health practice, program evaluation, or any other reason. The PRA also addresses customer satisfaction inventories, focus group inquiries, all types of surveys, telephone interviews, and electronic environments. One notable exception to the PRA is that federal employees may be solicited for information if the solicitation is in their line of work and provides information that is relevant to their work experience (10).

## Office of Management and Budget (OMB) Clearance

When the information gathering intended for evaluation meets the parameters specified in the PRA, the federal agency sponsoring the data collection applies for review and approval before the data collection begins. The U.S. Office of Management and Budget (OMB) oversees all requests for review and approval under the PRA (10). The agency sponsoring the data collection must submit an OMB clearance package explaining and justifying the data collection. In addition, two notices with subsequent 30- and 60-day comment periods must be published in the *Federal Register,* a daily U.S. government publication of the National Archives and Records Administration. OMB clearance typically takes 6 to 9 months (10). However, some OMB clearance processes take more than 12 months, partially because of the overwhelming number of submissions and limited number of staff members (9). To compensate for the PRA requirements and time required to obtain OMB clearance, evaluation plans should begin months before data collection begins.

Although the time involved in obtaining OMB clearance can pose a considerable challenge to evaluators, the process can also significantly improve the quality of the data collection. Program planners and evaluators should work together to focus their efforts and use ethical data-collection methods to obtain useful and necessary data. Such cooperation improves the program results while decreasing the public's burden.

During the clearance process, OMB requires detailed descriptions of the following (10):

1. The reasons the data collection is necessary
2. The purpose and use of the information that will be obtained from the data collection
3. The use of improved information technology to reduce burden
4. Efforts to identify the duplication and use of similar information
5. The possible impact of the data collection on small businesses or other small entities (e.g., other people or groups)
6. The consequences of collecting the data less frequently than planned
7. Special circumstances relating to Title 5, Part 1320.5 ("Controlling Paperwork Burdens on the Public") of the Code of Federal Regulations (CFR) guidelines about federal information collections
8. Comments in response to the *Federal Register* notice and evidence of efforts to consult with individuals other than those in the agency collecting the information
9. An explanation of any payments or gifts to respondents
10. Confidentiality assurances provided to respondents
11. Justification for sensitive questions
12. An estimate of annualized cost and burden hours
13. Annualized government costs 
14. An explanation for any program changes or adjustments
15. A plan for tabulation and publication and a time schedule
16. Any reasons that an OMB expiration date might be inappropriate to post on instrumentation (i.e., all PRA surveys and materials)
17. Exceptions to certification for the PRA submission

## Conclusion

Careful attention to the requirements for OMB clearance enhances program efficiency and improves evaluation processes and outcomes. The requirements are consistent with academic and professional recommendations that program planning be carried out with evaluation in mind; the two processes need to be simultaneous for best results (3,11).

The general public recognizes that the OMB review and approval process is a positive procedure that holds the federal government accountable for the public's burden to provide data. Federal agencies that are striving to provide evidence that tax dollars are being well spent consider OMB compliance to be a difficult but necessary obstacle to overcome. The PRA, which is enforced by the OMB, is a reasonable compromise between reducing paperwork burdens on the public and maximizing the benefits of data collection to ensure that well-planned public health programs have meaningful evaluations. Knowledge about and compliance with the PRA requirements are the essential component of the compromise, improving taxpayer satisfaction and government accountability. Fortunately, all necessary information about the PRA and OMB is available online from www.whitehouse.gov/omb/inforeg/infocoll.html, which guides program planners and evaluators through the process.
